# Fairy wrasses perceive and respond to their deep red fluorescent coloration

**DOI:** 10.1098/rspb.2014.0787

**Published:** 2014-07-22

**Authors:** Tobias Gerlach, Dennis Sprenger, Nico K. Michiels

**Affiliations:** Animal Evolutionary Ecology Group, Faculty of Sciences, University of Tübingen, Auf der Morgenstelle 28 E, Tübingen, Germany

**Keywords:** red fluorescence, marine visual ecology, signalling, visual communication, private channel, fish coloration

## Abstract

Fluorescence enables the display of wavelengths that are absent in the natural environment, offering the potential to generate conspicuous colour contrasts. The marine fairy wrasse *Cirrhilabrus solorensis* displays prominent fluorescence in the deep red range (650–700 nm). This is remarkable because marine fishes are generally assumed to have poor sensitivity in this part of the visual spectrum. Here, we investigated whether *C. solorensis* males can perceive the fluorescence featured in this species by testing whether the presence or absence of red fluorescence affects male–male interactions under exclusive blue illumination. Given that males respond aggressively towards mirror-image stimuli, we quantified agonistic behaviour against mirrors covered with filters that did or did not absorb long (i.e. red) wavelengths. Males showed significantly fewer agonistic responses when their fluorescent signal was masked, independent of brightness differences. Our results unequivocally show that *C. solorensis* can see its deep red fluorescent coloration and that this pattern affects male–male interactions. This is the first study to demonstrate that deep red fluorescent body coloration can be perceived and has behavioural significance in a reef fish.

## 1. Introduction

Colour signals appear particularly strong if they involve wavelengths that are otherwise missing from the environment. Which colours can be displayed, however, depends on the prevailing ambient light conditions. A particularly striking constriction of the available spectrum occurs in marine habitats, where the low-energy, long-wavelength part of the downwelling sunlight (more than 600 nm) is quickly absorbed by seawater, leaving little red and orange light below 10–20 m depth [1–3]. Therefore, in all but the shallowest euphotic environments, red pigments of marine fish cannot reflect red light and will appear dark grey [4–7]. This wavelength-specific attenuation of sunlight is accompanied by a dominance of blue and yellow body colours in reef fishes [8]. Consistent with these prevailing hues, the visual systems of most reef fish investigated to date have spectral sensitivities biased towards short and intermediate wavelengths [9,10]. As a consequence, previous research on reef fish vision has focused on the 350–600 nm range of the colour spectrum [3].

The recent discovery of red fluorescent coloration in more than 180 fish taxa has, however, challenged this view [11,12]. In contrast to the prevalent reflective coloration, fluorescent pigments absorb short-wavelength light and re-emit photons at longer wavelengths. As a consequence, fluorescence can generate red colour even when the corresponding long wavelengths are entirely absent from the ambient light environment. Thus, fluorescent pigments may offer fish the opportunity to generate conspicuous colour contrasts [6,11], particularly in deeper waters.

Measurements of the spectral sensitivity of the goby *Eviota atriventris* (formerly *Eviota pellucida* [13]) have shown that this species possesses long-wavelength visual pigments that make it physiologically sensitive to this species’s red fluorescent coloration [11]. Moreover, fluorescent particles can be actively aggregated and dispersed within specialized chromatophores via hormonal and nervous control [7,14], corroborating the proposed role of red fluorescence as a signalling mechanism in reef fish [11]. While fluorescence has been associated with visual signals in parrots [15,16], spiders [17] and mantis shrimps [18], experimental data illustrating any behavioural response to fluorescent colour stimuli in reef fishes are lacking to date and the ecological role of long-wavelength fluorescence remains to be shown [6].

**Figure 1.**
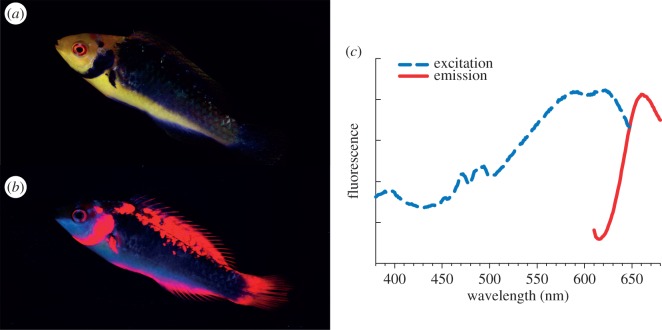
Fluorescence characterization of *C. solorensis*. (*a*) Male fish illuminated with broad-spectrum white light; (*b*) same individual under monochromatic blue illumination. (*c*) Excitation (dashed line) and emission (solid line) spectra of opercular scales. (Online version in colour.)

Here, we study behavioural responses elicited by red fluorescent colour patterns in the fairy wrasse *Cirrhilabrus solorensis* [19]. The genus *Cirrhilabrus* comprises more than 40 closely related species of small, diurnal Indo-Pacific labrids [20]. Fairy wrasses are common at the base of reef slopes at depths between 10 and 65 m [21,22], well below the depth to which red sunlight can penetrate. *Cirrhilabrus solorensis* features distinct red fluorescent body coloration (figure 1) with a unique deep red peak emission around 660 nm. Fluorescent emission in a comparable wavelength range has to date only been documented in one other reef fish species, the wrasse *Pseudocheilinus evanidus* [11]. Our own measurements show that other species of wrasses (for example in the genera *Paracheilinus* and *Symphodus*) also show deep red fluorescence (T.G. & N.K.M. 2013, unpublished data). In deep-sea dragon fishes, deep red fluorescence has been associated with bioluminescence [23,24], which has been proposed to constitute a private waveband used for interspecific communication and prey illumination ([25,26] and references therein). For marine fish living in the euphotic zone, however, the ability to perceive such deep (more than 650 nm) red colours has never been shown.

In this study, we test the hypothesis that *C. solorensis* can perceive its own deep red fluorescence and demonstrate the behavioural significance of fluorescent colour patterns in intraspecific interactions. We chose a behavioural response assay as our experimental paradigm in order to capture the synthesis of all sensory and neural processes while also providing indications for adaptive significance [27]. In the field, males court groups of females while defending their territories against other males. Pilot experiments in the laboratory showed that male *C. solorensis* react towards their own mirror image with threat displays, chasing and biting in ways similar to the behaviour shown in male–male interactions in the field (T.G. 2011, personal observation). Such mirror-image stimuli (MIS) are commonly used in studies of fish ethology and enable the experimental manipulation of colour and illumination level via filters (reviewed in [28]). Here, we quantified agonistic reactions of males confronted with a set of MIS treatments that either showed or concealed the red fluorescent component of the mirror image, supplemented by control treatments with different brightness.

## 2. Material and methods

### (a) Study species

The fairy wrasse *C. solorensis* was selected as a study species due to its deep red fluorescent body pattern, its occurrence at depths devoid of red sunlight and its display of diverse intrasexual behaviour. Being protogynous hermaphrodites [29], all terminal-phase males are derived from initial-phase females. *Cirrhilabrus solorensis* exhibits a strong dimorphism between these successive sexual phases: males are generally larger and have longer pelvic fins than females (see also [30]), but most notably feature a distinct body pattern that appears purple under broad-spectrum white light but fluoresces red under monochromatic blue light illumination (figure 1).

### (b) Animal maintenance

Experiments were conducted in the laboratory at the University of Tübingen, Germany, between September 2012 and January 2013, and approved by the local state authority under permit no. ZO 1/12. A total of 27 adult male individuals were obtained from an ornamental fish trader (von Wussow Importe, Pinneberg, Germany) and housed individually in 60 l aquaria. Opaque black PVC sheets between tanks were used to prevent males from seeing each other and thus avoid uncontrolled agonistic interactions. Each aquarium contained a small flower pot as shelter. All fish were fed daily with a standardized mixture of *Mysis* shrimp and *Calanus* zooplankton. Water was kept at a temperature of 25–26°C and 33–35 ppt salinity. Illumination was set to a 12 L : 12 D cycle. To confine red colour to fluorescence and to exclude interfering ambient red light, all animals were kept and experimentally tested under nearly monochromatic blue illumination (LED spots no. 71104, Lumitronix GmbH, Hechingen, Germany). Neither ultraviolet (less than 400 nm) nor wavelengths of more than 520 nm were present in the illumination spectrum, which featured a peak emission (*λ*_max_) of 462 nm, a predominant wavelength in clear oceanic waters [31].

Prior to testing the effects of red fluorescence on territorial defence reactions, all fish were acclimatized to their tanks for a minimum of 45 days to ensure that the fish had accustomed well and successfully established new territories in their respective aquaria. Eleven males failed to do so—these individuals turned out to be highly timid, and persistently concealed themselves upon the appearance of the experimenter and during any subsequent treatment. As this rendered behavioural observations towards a mirror image impossible, those fish were excluded from further experimentation. Each of the 16 remaining male fish was repeatedly exposed to every experimental treatment.

### (c) Experimental treatments and filter properties

In order to test the effects of red fluorescent body coloration on male agonistic behaviour, we presented individual *C. solorensis* with a 15 × 15 cm silver glass mirror and manipulated the colour composition of the mirror image by covering the mirror with different colour filters (LEE Filters, Hampshire, UK) held in place by metal pegs.

**Figure 2.**
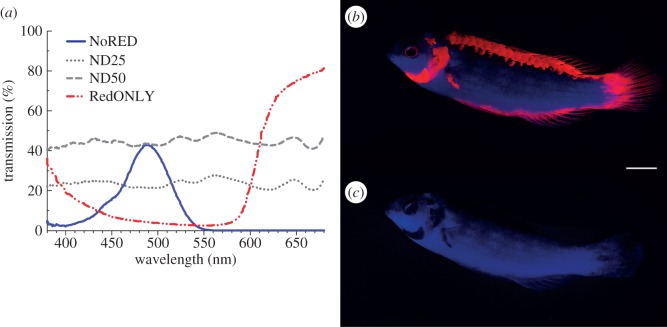
Qualitative transmission of filters. (*a*) Transmission spectra of the filters used. (*b*) Male fish photographed through filter ND25 under experimental light (white scale bar is 1 cm); (*c*) same individual photographed through filter NoRED. Both pictures were taken with a short-wavelength-reducing filter (see ‘Fluorescence photography and morphometric parameters’ section for details). (Online version in colour.)

In the experimental treatment (NoRED), a red-opaque filter (LEE no. 729, figure 2*a*,*c*) was used in front of the mirror to block all wavelengths between 550 and 750 nm. With such a filter, the mirror reflects fish in the ambient blue colours while masking its red fluorescence. As this filter not only blocks red light but also decreases brightness in the blue–green spectrum, we needed to rule out the possibility that male wrasses display less agonistic behaviour towards a non-red mirror image simply because it appears darker. For this reason, we used two different neutral density (ND) filters as controls. These ND filters (LEE no. 209 and no. 210) alter brightness independent of hue (i.e. they transmit all colours—including red—but reduce the overall brightness of the mirror image to 50% and 25% of the ambient light, respectively; control treatments ND50 and ND25, figure 2*a*,*b*). As a positive control, we presented the fish with a mirror (control treatment NoFILTER), which generated a bright mirror image containing all available wavelengths, including the fish's red fluorescence.

To examine whether the red fluorescent patches alone elicit any behavioural response, we also covered the mirror with a filter that blocks the wavelength range 380–600 nm (LEE no. 106; figure 2), thus transmitting only the red fluorescent body pattern while obscuring the blue reflection of the fish and so dissociating the colour patch from the fish shape (control treatment RedONLY). In order to ensure that all the agonistic behaviour observed was caused by the mirror image and not by the mere presence of the glass pane or filter sheet, we added several negative controls: each filter was also presented separately against the grey, non-reflective back of the mirror (negative control treatments back + NoRED, back + ND50, back + ND25, back + RedONLY). To further eliminate possible olfactory and chemical cues, all filters used in this experiment were present in the aquaria simultaneously during each treatment, concealed at the reverse side of the mirror.

Preliminary analyses showed that in all these negative controls, as well as in the treatment only transmitting red fluorescent coloration without the outline of the fish (RedONLY), the fish showed no aggressive behaviour. In order to focus on *planned comparisons* and reduce the risk of type I errors [32], we excluded these control treatments from further statistical analysis.

Qualitative filter transmission characteristics (figure 2*a*) were measured with a spectrometer (QE65000, Ocean Optics, FL), connected via a fibre-optic cable (Ocean Optics QR600–7-UV125BX) to a halogen light source (Ocean Optics HL-2000), with the light-emitting and -collecting probe pointing at a diffuse white reflectance standard (Spectralon SRS-99, Labsphere, NH). Filters were placed individually in an in-line filter holder (Ocean Optics FHS-UV) in the light path leading to the spectrometer, and transmission data were recorded with Spectra Suite v. 6.1 software (Ocean Optics).

**Figure 3.**
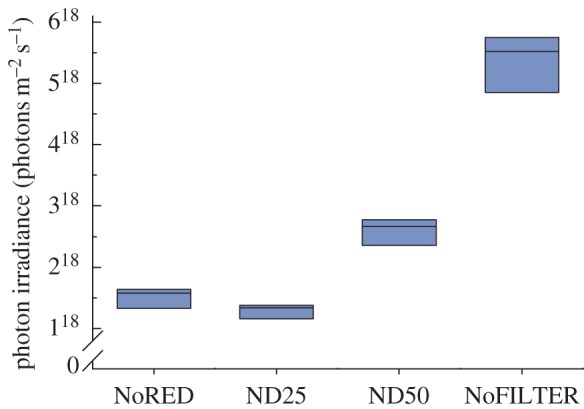
Quantitative transmission of filters. The graph shows the total amount of light transmitted through each filter under the experimental light conditions (*n* = 5 measurements per filter). (Online version in colour.)

To also assess quantitative transmission properties of the filters (figure 3), we measured the overall amount of light (380–780 nm) transmitted under experimental conditions using a portable photospectrometer (SpectraScan PR-670 with Cosine Corrector CP-670, Photo Research Inc., CA). With the filter completely covering the spectrometer's photo detector, we took five standardized measurements of photon irradiance for each filter used.

### (d) Experimental procedure and data recording

For each single treatment, a mirror with attached filters was carefully lowered into the water and placed at the side of the tank, whereupon the experimenter withdrew to minimize human interference. The fish's behaviour was then recorded for 2 min with a video camera (Sony HDR-CX6) mounted on a tripod parallel to the mirror pane. Experimental testing started in the morning and finished in the early afternoon. To eliminate daytime as a confounding factor, the testing sequence was designed in such a way that each day we started with a different animal, which was then subjected to all treatments in a randomized sequence; the completion of such a sequence was termed an experimental run. All animals underwent five experimental runs conducted on consecutive days, which resulted in a total number of 45 observations for each individual. Owing to constraints in laboratory space, the experiment had to be divided into two sequential trials with eight animals each.

Behavioural data were extracted from the video sequences with the observer always blind to the treatment (see electronic supplementary material). We evaluated the frequency of three distinct agonistic behaviours: (i) display, (ii) bite and (iii) tail-slap. Display behaviour was initiated by the fish swimming parallel to the mirror, whereupon the animal abruptly stopped and erected all fins before swimming on again. Bites were counted each time the fish bit the mirror, which sometimes culminated in attempted jaw locking. Tail-slaps consisted of a sudden hitting motion of the labrid's caudal fin against the mirror and were usually observed at the end of a sequence of agonistic reactions.

### (e) Fluorescence photography and morphometric parameters

One week after completion of the behavioural experiment, we measured individual body length, total body area and red fluorescent body area of each fish. For this purpose, each individual was transferred into a small, custom-made aquarium with a scale bar. Fish were photographed under monochromatic blue illumination provided by two blue LED torches (mini compact LCD, Hartenberger, Köln, Germany), each in combination with a subtractive dichroic blue filter (FD2C, Thorlabs, NJ). We used a digital still camera (Canon EOS 7D), standardized settings (1/15th sec, f/8, ISO 800 and white balance of 7450 K) and an EF-S 60 mm f/2.8 macro lens in combination with an optical long-pass filter attenuating short wavelengths below 550 nm (LEE filter no. 105). The latter served to artificially enhance the visibility of the red fluorescent pattern for image analysis. The fish pictures were imported, calibrated and measured in ImageJ v. 1.45s [33]. For the fluorescent area measurements, we set the colour threshold function to select only pixels with RGB red values exceeding 210.

Fluorescence excitation and emission characteristics (figure 1*c*) were determined by measuring male opercular scale samples with a spectrofluorometer (QuantaMaster 40, Photon Technology International, NJ) equipped with two liquid light guides (LLG 380): one for excitation aimed at a 45° angle at the fish scale and one for collection emission signal, aimed perpendicular to the scale. Both tips were less than 5 mm away from the sample. The sample was measured in salt water to limit osmosis-related artefacts and suppress reflection, which is much stronger in air. For this purpose, the tips of both light guides were also submerged. Excitation was varied from 330 to 730 nm in 4 nm steps. Emission was measured from 350 to 750 nm, also in 4 nm steps. The entry and exit slit of both monochromators (excitation source and emission measurement) was set to 5 nm. Emitted light was integrated by a photomultiplier (Hamamatsu PMT R928) in 1 s bins. The results were corrected for the transmission properties of the liquid light guides as well as the quantum efficiency of the photomultiplier at each measured wavelength.

### (f) Statistical analysis

Statistical analysis was done in R v. 2.15.2 [34]. Generalized linear mixed models (GLMMs) were used to examine sources of variation in the total number of displays, bites and tail-slaps between treatments. All response variables represented count data following a Poisson distribution and were modelled using the glmer function in the ‘lme4’ package [35]. To account for repeated measurements per individual fish, all models contained *individual ID* as a random intercept factor with 16 levels. Fixed factors included the experimental *treatment* (four levels: treatment NoRED, controls ND25 and ND50, and positive control NoFILTER) as well as the *experimental trial* (two levels: first and second). After correcting for overdispersion, model reduction showed that the fixed factor *experimental trial* did not improve model fit as evaluated by the Bayesian information criterion. This indicates that the treatment effects did not differ between the two experimental trials, and we thus omitted this factor from the final analysis. Cases in which a given behaviour was not observed were included as zero values. One individual performed so many bites that it was considered an outlier and removed from the analysis. We conducted post hoc comparisons for each pair of treatments with Tukey's HSD, using the glht function in the ‘multcomp’ package [36]. In order to investigate potential effects of morphometric parameters on agonistic behaviour, we added total body length, total body area, red fluorescent body area and all their interactions as additional covariates to the model. All results were considered significant at *p* < 0.05.

## 3. Results

**Figure 4.**
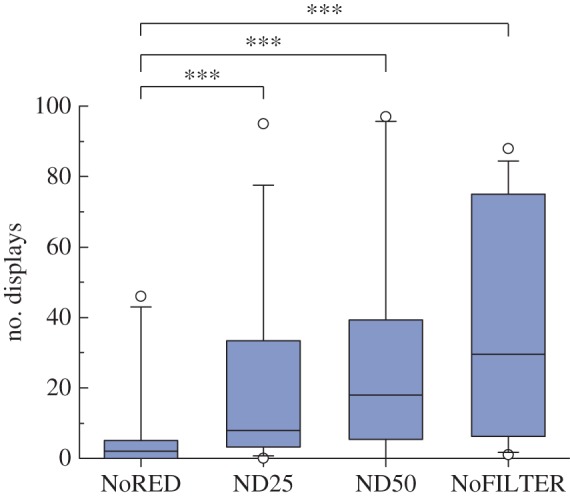
Total number of display behaviours under different treatments (*n* = 16 fish**)**; ***p < 0.001. (Online version in colour.)

**Figure 5.**
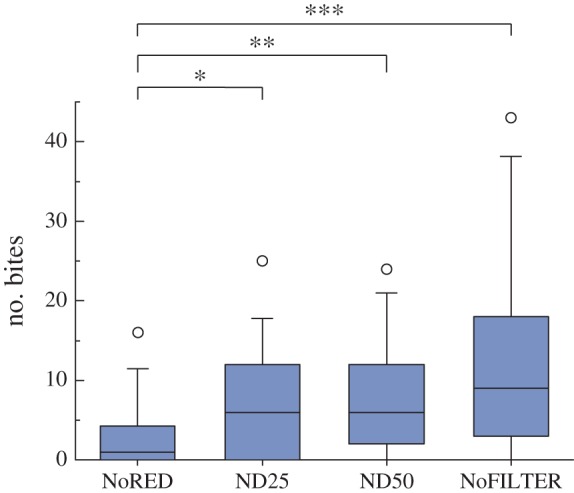
Total number of bites under different treatments (*n* = 15 fish); *p < 0.05, **p < 0.01, ***p < 0.001. (Online version in colour.)

We observed significantly less display behaviour under the experimental NoRED treatment compared with controls ND25, ND50 and NoFILTER (Tukey's HSD tests, all *p* < 0.001; figure 4). Bites were also significantly less frequent in the NoRED treatment compared with all control treatments, while we found no significant difference between the different control treatments (table 1 and figure 5). Tail-slaps were observed too rarely to make a statistical analysis meaningful.

**Table 1.** Pairwise comparisons of the effects of treatment on the total number of bites (Tukey's HSD; *n* = 15 fish).

treatment pair	estimate	s.e.	*z*-value	*p*
NoRED versus ND25	−1.0651	0.3982	−2.675	0.037
NoRED versus ND50	−1.2736	0.3938	−3.234	0.006
NoRED versus NoFILTER	−1.7458	0.3880	−4.500	<0.001
ND25 versus ND50	−0.2085	0.3548	−0.588	0.935
NoFILTER versus ND50	0.4722	0.3404	1.387	0.506
NoFILTER versus ND25	0.6807	0.3477	1.958	0.203

The direct comparison between the experimental NoRED treatment and the darkest control (ND25) is particularly revealing: under our experimental light conditions, the NoRED filter transmits approximately 15% more light than the red-transparent control filter ND25 (figure 3). Nevertheless, both displays and bites occurred significantly less frequently under the NoRED treatment compared with the ND25 treatment (display behaviour: *z* = −4.559, *n* = 16, *p* < 0.001, Tukey's HSD; bites: *z* = −2.675, *n* = 15, *p* = 0.034).

The morphometric parameters body size, total body area and fluorescent body area, and all of their interactions, did not have statistically significant effects on the observed agonistic behaviours (GLMMs for morphometric parameters, all *p* > 0.25).

## 4. Discussion

Male *C. solorensis* showed significantly fewer agonistic responses when confronted with a mirror image masking their red fluorescent body patterns compared with control treatments where their fluorescent coloration remained visible. Pairwise comparisons between control treatments revealed that a change in brightness alone had no significant effect on the observed behaviour. This clearly suggests that agonistic behaviour in *C. solorensis* is influenced by the presence of red fluorescent body coloration in the fish's mirror image, rather than through a change in brightness.

We thus conclude that (i) *C. solorensis* is able to perceive the deep red fluorescent coloration of its conspecifics and that (ii) this fluorescent colour pattern affects agonistic male–male interactions. This is the first study to demonstrate that deep red fluorescent body coloration can have a behavioural significance in a reef fish.

Why does the red fluorescent coloration influence male agonistic interactions? One explanation is that this colour pattern facilitates the recognition of male conspecifics, similar to the role of purely reflective colour patterns in other marine and freshwater fish [37–39]. An experimentally manipulated mirror image lacking that stimulus could therefore fail to be recognized as a rival. However, the fact that males did show some agonistic behaviour when confronted with a red-deprived mirror image—although at significantly lower rates—indicates that even without red colour, the mirror image was perceived as a potential intruder. Also, when protogynic *Cirrhilabrus* wrasses change sex from initial-phase females to terminal-phase males, transitional-phase individuals already resemble males in shape but still lack the fluorescent dorsal and opercular stripe (T.G. 2011, personal observation; see also [30,40]). A red-deprived mirror image may therefore be perceived as a transitional male that is not yet judged as a fully competent rival, and thus receives only limited attention by territorial males.

The mere presence of deep red colour without the outline of the fairy wrasse (treatment RedONLY) proved insufficient to evoke any aggressive responses. This is not unexpected because many other reef organisms (such as stony corals and calcareous algae) also exhibit red fluorescence [11].

In recent years, short-wavelength ultraviolet colour patterns have been shown to serve species recognition and modulate male aggression in damselfish [39,41], and affect mate choice and territorial behaviour in guppies [42] and sticklebacks [43–45]. As many predatory fish are unable to detect ultraviolet light [46,47], UV coloration has been suggested to act as a private communication channel [41,48]. Red fluorescence in reef fish also has the potential to serve private communication: the fluorescent colour pattern of *C. solorensis* peaks at around 660 nm, a visual range for which most reef fish families have poor or no sensitivity [3,10,49]. This reduced sensitivity for red probably represents an adaptive response to the lack of long-wavelength sunlight in most marine habitats, making its perception superfluous. In this blue-dominated environment, however, red fluorescence enables fish to display signals with a particularly high chromatic contrast and conspicuousness to those few receivers that possess photoreceptor sensitivity in this long-wavelength range. The same signals remain invisible (or at least inconspicuous) to others with peak sensitivities at shorter wavelengths.

The suitability of red colour signals for private communication is further enhanced by the rapid attenuation of long wavelengths in seawater [2]: red fluorescent coloration is particularly well suited for short-range visual interactions, as is usually the case for social and sexual interactions among conspecifics. At the same time, its information content is rapidly lost at the greater distances relevant for most predators to detect their prey. As this study demonstrates that fairy wrasses do perceive their fluorescent colour pattern and use it for intraspecific interactions, we propose that *C. solorensis* may have shifted its visual communication towards wavelengths that predatory fish are less likely to pick up.

Our discovery of a reef fish that uses long-wavelength fluorescence for intraspecific interaction raises several questions that will be addressed in future work: first, physiological characterizations of the long-waveband photoreceptor sensitivity of these fish will help towards understanding the intermediate perceptual steps enabling the behavioural responses documented here. Second, the fluorescent pigment and its associated costs should be characterized. Third, in addition to the male–male interactions described here, the male-limited fluorescent pattern of *C. solorensis* is a good candidate trait to affect female choice. Finally, to investigate the potential use of red fluorescence as *private communication*, the exact visual capabilities of predators in this wavelength range need to be examined, while taking into account functional costs of evolving the ability to detect such signals [49].

## Supplementary Material

Behaviour dataset
